# A Metabolic Study of Huntington’s Disease

**DOI:** 10.1371/journal.pone.0146480

**Published:** 2016-01-08

**Authors:** Rajasree Nambron, Edina Silajdžić, Eirini Kalliolia, Chris Ottolenghi, Peter Hindmarsh, Nathan R. Hill, Seán J. Costelloe, Nicholas G. Martin, Vincenzo Positano, Hilary C. Watt, Chris Frost, Maria Björkqvist, Thomas T. Warner

**Affiliations:** 1 Department of Clinical Neurosciences, UCL Institute of Neurology, London, United Kingdom; 2 Brain Disease Biomarker Unit, Department of Experimental Medical Science, Wallenberg Neuroscience Centre, Lund University, Lund, Sweden; 3 APHP, Department of Metabolic Biochemistry, Necker Hospital, Paris, France; 4 Developmental Endocrinology Research Group, UCL Institute of Child Health, London, United Kingdom; 5 Nuffield Department of Primary Care Health Sciences, University of Oxford, Oxford, United Kingdom; 6 Department of Clinical Biochemistry, Royal Free London NHS Foundation Trust, London, United Kingdom; 7 Fondazione CNR-Regione Toscana G. Monasterio, Pisa, Italy; 8 Department of Public Health and Primary Care, Imperial College, London, United Kingdom; 9 Department of Medical Statistics, London School of Hygiene and Tropical Medicine, London, United Kingdom; 10 Reta Lila Weston Institute of Neurological Studies, UCL Institute of Neurology, London, United Kingdom; GDC, GERMANY

## Abstract

**Background:**

Huntington’s disease patients have a number of peripheral manifestations suggestive of metabolic and endocrine abnormalities. We, therefore, investigated a number of metabolic factors in a 24-hour study of Huntington’s disease gene carriers (premanifest and moderate stage II/III) and controls.

**Methods:**

Control (n = 15), premanifest (n = 14) and stage II/III (n = 13) participants were studied with blood sampling over a 24-hour period. A battery of clinical tests including neurological rating and function scales were performed. Visceral and subcutaneous adipose distribution was measured using magnetic resonance imaging. We quantified fasting baseline concentrations of glucose, insulin, cholesterol, triglycerides, lipoprotein (a), fatty acids, amino acids, lactate and osteokines. Leptin and ghrelin were quantified in fasting samples and after a standardised meal. We assessed glucose, insulin, growth hormone and cortisol concentrations during a prolonged oral glucose tolerance test.

**Results:**

We found no highly significant differences in carbohydrate, protein or lipid metabolism markers between healthy controls, premanifest and stage II/III Huntington’s disease subjects. For some markers (osteoprotegerin, tyrosine, lysine, phenylalanine and arginine) there is a suggestion (p values between 0.02 and 0.05) that levels are higher in patients with premanifest HD, but not moderate HD. However, given the large number of statistical tests performed interpretation of these findings must be cautious.

**Conclusions:**

Contrary to previous studies that showed altered levels of metabolic markers in patients with Huntington’s disease, our study did not demonstrate convincing evidence of abnormalities in any of the markers examined. Our analyses were restricted to Huntington’s disease patients not taking neuroleptics, anti-depressants or other medication affecting metabolic pathways. Even with the modest sample sizes studied, the lack of highly significant results, despite many being tested, suggests that the majority of these markers do not differ markedly by disease status.

## Introduction

Huntington’s disease (HD) is a devastating hereditary neurodegenerative disorder characterised by progressive motor, cognitive and psychiatric impairment [1]. In recent years it has become clear that HD can be regarded as a systemic disorder affecting many organs and tissues causing peripheral as well as brain pathology [2]. Both animal and human studies indicate that some of the peripheral symptoms of HD, including weight loss and alterations in appetite, could be linked to endocrine and metabolic alterations [3, 4]. These alterations may be reflected in plasma levels of carbohydrate, lipid or protein metabolites and/or hormones related to energy metabolism.

### Carbohydrate metabolism

Studies of carbohydrate metabolism in patients with HD have generated ambiguous and conflicting results. Metabolic profiling of serum samples has shown significant changes in various monosaccharide levels, particularly glucose, between HD gene carriers and controls [5] and studies have shown impaired glucose tolerance and increased prevalence of diabetes in HD patients [6–8]. Insulin sensitivity studies in HD patients have shown both a decrease in insulin sensitivity and impaired insulin secretion capacity in normoglycemic subjects [9]. However, other studies in HD patients have reported normal glucose and insulin levels following a glucose tolerance test, as well as normal fasting glucose and insulin [10–15].

### Lipid metabolism

HD patients display changes in body fat stores, as indicated by decreased visceral and peripheral adiposity [15–17]. In addition, altered fatty acid metabolism and changes in various markers of fatty acid breakdown have been reported in HD [5, 18]. Dysfunction of the cholesterol biosynthetic pathway has also been shown in HD [19, 20] and cholesterol precursors and metabolites have been shown to be reduced in manifest HD patients [21, 22]. However, studies measuring total cholesterol concentration in plasma have reported both low [23, 24] and normal levels [21] in HD patients compared to healthy controls.

### Protein metabolism

Muscle wasting is a common feature of HD [15–17] and abnormal *in vivo* skeletal muscle energy generation has been shown in both symptomatic patients with HD and presymptomatic mutation carriers [25–28]. Several studies of amino acid metabolism in patients with HD have found a decrease in the concentrations of neutral amino acids (especially alanine, valine, leucine and isoleucine) in HD plasma [5, 14, 18, 29–34].

### Gastric and adipose hormones

Ghrelin, an orexigenic peptide of gastric origin, and leptin, a peptide hormone secreted by adipose tissue, are two peripherally produced hormones that exert effects on the hypothalamus in the regulation of body energy homeostasis [35]. Leptin induces weight loss by suppressing food intake and stimulating metabolic rate, whereas ghrelin stimulates appetite and increases adiposity [36, 37]. Studies on leptin in HD patients have reported similar concentrations in HD patients and controls [38, 39]. Conversely, it has also been reported that patients with HD have increased ghrelin and decreased leptin concentration in plasma compared with healthy controls [14, 24, 40].

### Aim of the study

The aim of this study was to evaluate a number of metabolic variables to assess whether they are linked to disease state in a cross-sectional study of cohorts of HD gene carriers and controls. We studied carbohydrate, lipid and protein metabolites as well as hormones related to energy metabolism in plasma samples from well-characterised cohorts of premanifest and moderate HD subjects and healthy controls.

## Materials and Methods

### Study participants

The study was approved by the joint UCL/UCLH ethics committee. Patients were eligible for enrollment if they were 18 years of age or older, had completed either a predictive test for premanifest subjects, or had a confirmed genotype consistent with HD (CAG repeat ≥40). Patients committed to undergo a 24-hour inpatient stay for the study and a body magnetic resonance imaging (MRI) scan. Controls were recruited principally from the partners, spouses, or caregivers of the HD group and exclusion criteria were the same as for the HD group.

Subjects were excluded if they had a history of alcohol or drug abuse in the preceding 12 months or if they had received medication in the preceding 6 months that could influence the hypothalamic-pituitary axis, such as corticosteroid treatment, antipsychotic medication including phenothiazine, or antiemetic drugs. Subjects could not have any pre-existent endocrine diseases, such as diabetes, or central nervous system disorder such as head trauma and seizures. Patients with metallic implants (contraindicated for MRI scan), those experiencing recent weigh loss or gain, and night shift workers were also excluded.

### Clinical protocol

The clinical protocol was described previously [41]. Study subjects were admitted to a private clinical room and had an intravenous cannula inserted. During the day the subject could walk freely, watch television, but not fall asleep or snack outside scheduled mealtimes. Scheduled meal times were: breakfast at 09.00, standardised test meal at 12:00 and dinner at 18:00. At 22:00 they retired to bed for sleep and lights were turned off. Lights were switched on again at 06:00. Hourly blood samples were taken over the 24-hour period using a long line from 14:00 to 13:00 the following day.

Clinical assessment and HD rating scales were performed by a neurologist with expertise in HD (TTW). Clinical assessment of patients was performed by taking the medical history and performing a whole body physical and neurological examination. The Unified Huntington’s Disease Rating Scale (UHDRS) was used to quantitatively measure HD signs [42–45]. Cognitive function was assessed by Stroop Test Evaluation Colour Naming (STECN), Stroop Test Evaluation Word Reading (STEWR), Stroop Test Evaluation Interference (STEI), Symbol Digit Test (SDT) and Verbal Fluency Test (VFT). Problem Behaviours Assessments were also performed.

Biometric data were obtained including body mass index (BMI), scapular fat thickness (SFT), waist-to-hip ratio (WHR) and circumference of the abdomen (CA). In addition, participants underwent a T1-weighted abdominal MRI scan in order to assess the visceral and subcutaneous adipose tissue using Hippofat software [46]. Visceral and subcutaneous adipose deposition was measured because, despite weight loss, enhanced accumulation of body fat in mid-life has been found in several HD mouse models [47–49].

### Collection and processing of human samples

Whole blood samples for lactate and plasma samples for glucose analysis were analysed on the day of collection: prior to analysis, lactate samples were kept on ice while glucose samples were kept at room temperature. Blood samples for insulin analysis were collected on ice and centrifuged immediately for 5 minutes at 2500 revolutions per minute (RPM) at 4°C, the serum collected, immediately placed on dry ice, and stored at -80°C until analysis. Blood samples for cortisol, growth hormone, leptin, ghrelin, total cholesterol, high-density lipoprotein (HDL)-cholesterol, low-density lipoprotein (LDL)-cholesterol and triglyceride analysis were allowed to clot at room temperature for 30 minutes, then centrifuged and stored as above. Blood samples for analysis of fatty acids, amino acids, β-hydroxybutyrate, lipoprotein (a), apolipoproteins and osteokines were immediately placed on ice, and centrifuged within 5 minutes of sampling, at 4°C at 2500 RPM for 5 minutes. Plasma was collected, immediately placed on dry ice and stored at -80°C until analysis. Leptin and ghrelin levels were assessed on two occasions: at 06:00 and 13:00, one hour after a standardised, 550 kcal meal comprising 30% lipids, 50% carbohydrates and 20% proteins [50, 51].

A 3-hour Oral Glucose Tolerance Test (GTT) was performed from 06:00 to 09:00 with blood samples taken just prior to and at 30 minutes intervals after ingestion of an oral glucose load of 75 grams. These samples were used for glucose, insulin, cortisol and growth hormone analysis. The homeostasis model assessment of beta-cell function and insulin resistance (iHOMA2) was used in default mode to calculate insulin secretion and sensitivity [52]. For each patient, any perturbation in glucose & insulin homeostasis was quantified using the Observed Variability and Lability (OVAL) model [53]. Diabetes was defined as fasting plasma glucose >7 mmol/L and/or 2 hour sample >11.2. Impaired glucose tolerance was defined as fasting plasma glucose <7 mmol/L and 2 hour sample between 7.8 and 11.1 mmol/L and impaired fasting glycaemia as fasting blood glucose between 6.1 mmol/L and 6.9 mmol/L and 2 hour sample <7.8 mmol/L.

### Sample analysis

Plasma glucose was assayed on the Roche Modular P Analyser using the glucose oxidase method, whereas whole blood lactate was measured on the Siemens Blood Gas Analyser. Serum insulin was analysed by a microparticle immunoassay run on an Abbott AxSYM Analyser. Serum cortisol concentrations were determined by electrochemiluminescent immunoassay on a Roche Modular E170 Autoanalyser. Serum growth hormone was quantified using a chemiluminescent immunoassay on a Siemens Immulite Analyser. Serum leptin was measured by an enzyme-linked immunosorbent assay (R&D Systems Europe, Abingdon, UK), while serum active ghrelin was measured by radioimmunoassay (Millipore, Billerica, MA, USA). The plasma amino acid profile was determined by reversed-phase partition high performance liquid chromatography (HPLC) and by ion exchange chromatography as previously described [14]. Osteokines and the bone-specific isoform of alkaline phosphatase were quantified using the Human Bone Panel I and II assays as per the manufacturer’s protocol (Meso Scale Discovery, Rockville, MD, US). Apolipoproteins and lipoprotein (a) were measured by immunonephelometry on a Siemens BN2 analyser. Total cholesterol, HDL and triglycerides were measured using enzymatic-spectrophotometric assays on a Roche Modular P Analyser. LDL concentration was calculated from the Total cholesterol, HDL and Triglyceride results using the Friedewald equation [54]. Serum leptin and ghrelin, and plasma amino acids and osteokines were measured in duplicate and readings averaged. All other variables were single measurements.

### Statistical Analysis

Inter-group differences were assessed using linear regression models with group, age and gender as predictor variables. Where residuals were not normally distributed, variables were logarithmically transformed and the analysis repeated. For all regression models joint F-tests were used to compare adjusted group means. A chi-square test was used to compare proportions of subjects with and without impaired glucose tolerance. The significance level was set at p<0.05. Statistical analyses were performed using SPSS for Windows (release 16.0, SPSS, Inc., Chicago, IL) and using Stata (StataCorp Stata Statistical Software: Release 13. College Station, TX: StataCorp LP).

## Results

Fifteen control subjects, 14 premanifest HD gene carriers, and 13 moderate (stage II/III) HD patients were enrolled into the study. Study participant characteristics are summarised in Table 1.

**Table 1:** Demographic and clinical characteristics of control and HD subjects.

Stage	Controls	Premanifest HD	Stage II/III HD
Number of subjects	15	14	13
Mean age (range)	52 (29–69)	45 (31–58)	55 (42–70)
Female:Male	6:9	9:5	5:8
Mean CAG (range)	-	42 (40–47)	44 (42–47)
Mean burden score (range)	-	301 (207–434)	435 (273–702)
Mean UHDRS Total Functional Capacity	-	13 (12–13)	8 (5–12)
Mean UHDRS Motor ScoreMean	-	2 (0–11)	38 (10–65)
BMI ± SD	26.0 ± 4.3	28.9 ± 4.7	25.7 ± 3.5
Mean scapular fat thickness ± SD (mm)	15.5 ± 7.2	22.2 ± 6.3**	17.5 ± 7.3*
Mean waist to hip ratio ± SD	0.87 ± 0.09	0.89 ± 0.08**	0.88 ± 0.06*
Mean abdominal circumference ± SD (cm)	92.1 ± 14.3	102.2 ± 14.0***	94.4 ± 11.0**
Subcutaneous adipose tissue ± SD (cm^2^)	158.4 ± 53.7*	265.3 ± 148.4****	176.7 ± 87.0
Visceral adipose tissue ± SD (cm^2^)	118.5 ± 92.8**	95.5 ± 59.7****	117.5 ± 56.9
Total fat ± SD (cm^2^)	281.8 ± 115.7**	360.8 ± 154.9****	294.2 ± 112.8
Body area ± SD (cm^2^)	605.6 ± 197.5*	690.8 ± 156.0****	620.2 ± 115.7
VAT/SAT ratio ± SD (cm^2^) (%)	79.6 ± 77.3**	51.2 ± 57.8****	77.5 ± 54.6
SAT %	44.2 ± 7.0**	50.5 ± 13.8****	46.3 ± 12.6

* One missing value

** Two missing values

*** Three missing values

**** Four missing values.

### Carbohydrate metabolism

#### Glucose tolerance test

**Fig 1:**
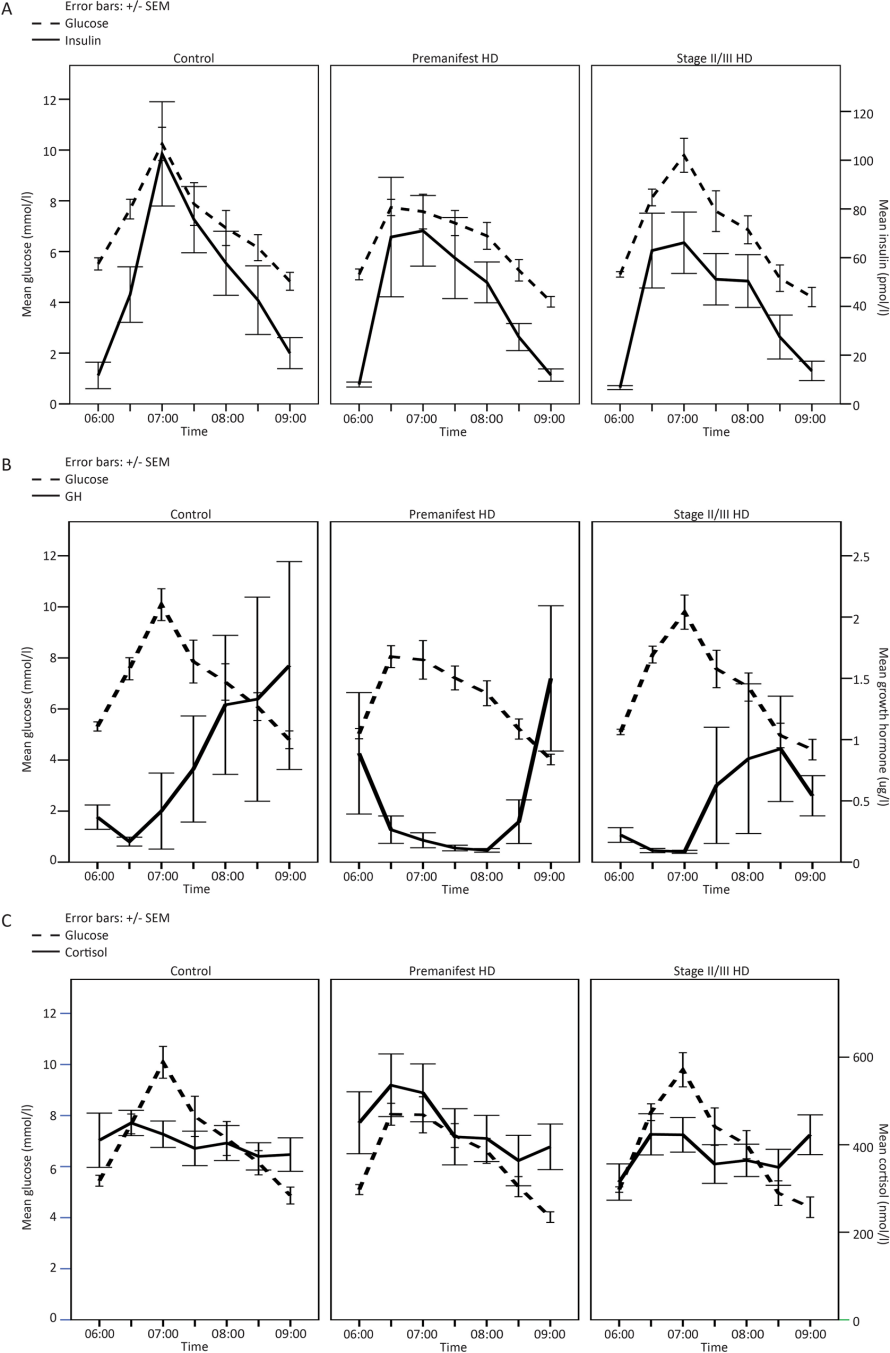
Mean blood glucose levels at different time points during a glucose tolerance test in controls, premanifest and moderate HD patients and the corresponding mean insulin (A), growth hormone (B) and cortisol (C) concentrations.

We performed a glucose tolerance test with blood sampling every 30 minutes over 3 hours and quantified glucose, insulin, cortisol and GH in these samples. No significant difference between control, premanifest and stage II/III HD subjects was found in baseline glucose, insulin, GH and cortisol concentrations, nor during the glucose tolerance test (Fig 1). Fig 1A and 1B suggests there may be a blunted insulin and GH response in stage II/III HD compared to healthy controls but this did not reach statistical significance. We used a chi-square test to compare proportions of subjects with and without impaired glucose tolerance. The proportion of subjects with glucose intolerance did not differ between controls, premanifest HD and stage II/III HD subjects (χ2 = 0.039, P = 0.981).

Table 2 shows the mean and SD of homeostatic model assessment (HOMA)-S and HOMA-β which are measures of insulin secretion and beta cell function respectively, as well as OVAL, a measure of fasting glucose-insulin homeostasis, in controls, premanifest and stage II/III HD patients. After adjustment for age and gender there was no significant difference in HOMA-S, HOMA-β or OVAL between the three groups. Higher HOMA-S and OVAL homeostasis was observed in premanifest HD subjects compared to control and stage II/III HD subjects and higher HOMA-β was observed in stage II/III HD compared to controls and premanifest HD subjects, however none reached statistical significance (Table 2).

**Table 2:** Fasting levels of metabolic markers in control, premanifest and stage II/III HD cohorts. Data are presented as Mean ±SD for normally distributed data and as median [minimum—maximum] for skewed data.

	Control (n = 15)	Premanifest HD (n = 14)	Stage II/III HD (n = 13)	*p*
**Carbohydrate metabolism**				
GTT–OVAL	317.97 ± 181.26	317.03 ± 139.00	440.78 ± 210.02	0.30
GTT–HOMA-S	282.14 ± 126.40	301.75 ± 93.71	378.47 ± 136.65	0.16
GTT–HOMA-β	45.20 ± 13.49	51.65 ± 21.67	43.35 ± 13.13	0.82
Lactate (mmol/l)	1.17 ± 0.26	1.30 ± 0.29	1.17 ± 0.41	0.39˄
**Bone markers:**				
Alkaline phosphatase, ng/ml	31.89 ± 8.28	36.12 ± 11.90	37.44 ± 10.83	0.38
Osteocalcin, ng/ml	42.64 ± 23.86	30.27 ± 11.58	39.88 ± 13.69	0.24
Osteonectin, ng/ml	121.6 [34.0–414.6]	167.5 [78.0–811.6]	122.2 [61.0–1340.2]	0.23*
Osteoprotegerin, ng/ml	0.20 ± 0.04	0.22 ± 0.04	0.21 ± 0.04	0.05
Sclerostin, ng/ml	0.25 ± 0.12	0.27 ± 0.12	0.26 ± 0.11	0.90
**Lipid metabolism**				
Total cholesterol (mmol/l)	4.69 ± 0.97	4.75 ± 1.03	4.57 ± 1.15	0.99
LDL cholesterol (mmol/l)	2.73 ± 0.83	3.01 ± 0.79	2.77 ± 1.04	0.86
HDL cholesterol (mmol/l)	1.46 ± 0.46	1.28 ± 0.39	1.35 ± 0.49	0.41
Triglycerides (mmol/l)	1.11 ± 0.45	1.18 ± 0.47	1.02 ± 0.47	0.41
Lipoprotein (a) (g/L)	0.17 ± 0.13	0.33 ± 0.28	0.20 ± 0.21	0.28
Apolipoprotein A1 (g/L)	1.40 ± 0.25	1.43 ± 0.31	1.35 ± 0.25	0.94
Apolipoprotein B (g/L)	0.77 ± 0.20	0.89 ± 0.21	0.82 ± 0.22	0.40
Fatty acids (mmol/l)	0.48 ± 0.21	0.41 ± 0.16	0.44 ± 0.20	0.55˅
B-hydroxybutyrate (mmol/l)	0.13 ± 0.09	0.07 ± 0.04	0.09 ± 0.06	0.09˄
**Hormones**				
Leptin (ng/ml), fasting	9.91 [4.38–27.44]	28.69 [2.39–116.22]	9.32 [2.22–52.02]	0.19*
Leptin (ng/ml), postprandial	8.70 [3.26–28.09]	28.29 [2.00–72.91]	8.48 [3.15–42.24]	0.19*
Ghrelin (pg/ml), fasting	57.90 [30.67–32.37]	77.07 [48.29–173.69]	58.85 [32.19–122.66]	0.05*
Ghrelin (pg/ml), postprandial	56.61 [27.46–51.73]	50.78 [39.97–133.87]	58.91 [30.87–131.18]	0.67*$

Unless otherwise indicated p-values are from linear regression models, comparing group means after adjustment for gender and age.

*p-value from linear regression of log transformed values, comparing group means after adjustment for gender and age.

˄Control (n = 13); Premanifest HD (n = 13); Stage II/III HD (n = 12)

˅Control (n = 14); Premanifest HD (n = 14); Stage II/III HD (n = 12)

$Control (n = 14); Premanifest HD (n = 14); Stage II/III HD (n = 13)

### Bone turnover markers

We investigated markers of bone turnover by measuring proteins released during bone formation and degradation products produced during bone resorption [55]. After adjustment for age and gender we did not observe a significant difference in bone formation markers bone alkaline phosphatase, oesteocalcin and osteonectin, nor in the inhibitor of bone formation, sclerostin, between control and HD subjects (Table 2). Between group differences in the bone resorption marker osteoprotegerin were statistically significant (p = 0.0497), but given the borderline nature of the p-value, the number of statistical tests performed (and corresponding increased risk of false positive findings), and the fact that the statistical significance was driven by adjusted levels in the pre-manifest being higher than those in the other two groups which were similar to one another, we are inclined to regard this as a chance finding.

### Lipid metabolism

In the current study we measured levels of total cholesterol (TC), low-density lipoprotein (LDL), high-density lipoprotein (HDL), triglycerides (TGS), apolipoprotein A1 (ApoA1), apolipoprotein B (ApoB), lipoprotein (a), fatty acids and B-hydroxybutyrate (or sodium 3-hydroxybutyrate). There was no significant difference in concentrations of any of the above variables between the three groups after adjustment for age and gender (Table 2).

T1 weighted abdominal MRI was used to quantitate visceral and subcutaneous adipose tissue using Hippofat software that automatically quantifies adipose tissue areas without user inputs [46, 56]. Adipose tissue characterisation in the three groups was similar (Table 1).

### Protein metabolism

#### Amino acids

We investigated fasting levels of amino acids, including branched chain amino acids (BCAAs, i.e. valine, leucine and isoleucine) in plasma samples using reversed-phase HPLC. After adjustment for age and gender statistically significant between group differences were found in phenylalanine, lysine and arginine concentrations (all three p-values between 0.02 and 0.05), although finding three such results is consistent with random chance given the number of variables investigated. In addition, in each case the statistical significance is driven by the fact that adjusted mean levels in the premanifest group were markedly higher than those in the stage II/III group and the controls, with mean levels in these two groups being similar, suggesting that this is not a biologically plausible finding. We did not find any significant between group differences in any of the other amino acids analysed (Table 3). We also quantified amino acid concentration using ion exchange chromatography as previously described [14]. After adjustment for age and gender only one variable (Tyrosine) differed significantly between groups, the result being only just statistically significant (p = 0.04) and again with adjusted levels highest in the premanifest group (Table 3).

**Table 3:** Protein metabolism. Levels of amino acids (μmol/L) in control, premanifest and stage II/III HD cohorts as determined by ion exchange chromatography and reversed-phase HPLC. Data are presented as Mean (SD).

Amino acid	Ion exchange chromatography				Reversed-phase HPLC			
	Control (n = 12)	Premanifest HD (n = 11)	Stage II/III HD (n = 12)	*p*	Control (n = 14)	Premanifest HD (n = 14)	Stage II/III HD (n = 12)	*p*
Taurine	137.3 (46.6)	127.9 (41.3)	141.5 (37.7)	0.91	70.3 (27.0)	78.4 (22.2)	65.0 (17.9)	0.16
Aspartic Acid	5.4 (3.2)	5.7 (3.7)	5.6 (1.5)	0.97	4.0 (2.2)	5.2 (2.8)	3.3 (1.2)	0.56
Threonine	144.8 (34.1)	146.7 (31.5)	141.6 (38.8)	0.53	139.4 (35.2)	123.1 (42.1)	134.7 (24.8)	0.59
Serine	127.0 (26.9)	127.8 (27.5)	126.8 (25.5)	0.99	118.7 (27.3)	125.7 (34.2)	115.0 (20.0)	0.68
Asparagine	62.8 (11.6)	64.1 (19.7)	59.5 (10.0)	0.57	57.0 (14.0)	57.8 (12.0)	50.0 (6.3)	0.19
Glutamic Acid	58.5 (30.0)	50.9 (19.8)	54.4 (24.8)	0.63	93.1 (81.2)	96.2 (62.4)	79.1 (36.9)	0.27
Glutamine	620.2 (97.4)	580.0 (87.1)	628.1 (71.1)	0.98	622.1 (146.0)	576.0 (86.0)	630.2 (70.2)	0.72
Proline	210.6 (48.3)	189.8 (62.8)	191.8 (63.3)	0.50	168.9 (54.7)	164.4 (37.7)	154.1 (44.7)	0.27
Glycine	268.8 (115.7)	282.5 (126.2)	275.6 (121.0)	0.99	246.6 (114.7)	245.9 (106.1)	243.7 (85.4)	0.99
Alanine	383.7 (151.0)	464.4 (126.2)	421.5 (100.6)	0.25	326.7 (123.7)	382.7 (55.3)	347.7 (84.9)	0.22
2-aminobutyric acid	31.5 (10.4)	28.7 (5.3)	30.3 (8.2)	0.63				
Citrulline	45.8 (13.2)	47.2 (17.2)	52.7 (17.1)	0.56				
Valine	259.9 (52.9)	278.1 (60.3)	260.7 (47.8)	0.06	231.2 (51.4)	241.3 (41.8)	233.8 (36.4)	0.17
Cysteine	109.1 (29.1)	95.7 (19.6)	107.3 (29.7)	0.91				
Methionine	24.3 (3.9)	25.5 (4.6)	25.3 (4.8)	0.24	24.4 (7.7)	26.2 (4.7)	24.0 (4.0)	0.19
Isoleucine	77.5 (21.2)	74.3 (18.0)	74.8 (18.0)	0.45	68.2 (20.5)	66.7 (12.5)	64.6 (13.8)	0.28
Leucine	134.8 (30.4)	140.7 (32.1)	139.7 (28.7)	0.11	127.6 (31.4)	134.3 (25.1)	129.2 (26.5)	0.09
Tyrosine	63.9 (15.8)	72.4 (11.3)	70.4 (8.9)	0.04	55.9 (16.4)	63.4 (12.1)	60.6 (9.4)	0.14
Phenylalanine	56.8 (10.0)	61.5 (5.9)	62.3 (10.7)	0.07	54.7 (11.0)	61.4 (7.0)	57.9 (8.4)	0.02
Ornithine	63.6 (13.7)	59.9 (20.0)	71.5 (23.7)	0.69	52.5 (13.9)	52.9 (16.6)	60.8 (15.0)	0.43
Histidine	76.9 (10.6)	78.5 (7.8)	82.3 (9.6)	0.47	83.4 (16.2)	90.6 (9.8)	84.8 (10.4)	0.25
Lysine	178.1 (34.8)	188.3 (36.8)	191.3 (31.6)	0.41	184.7 (40.8)	209.4 (29.1)	186.8 (33.1)	0.02
Arginine	85.9 (16.3)	93.1 (18.7)	98.1 (21.0)	0.14	87.3 (14.9)	106.8 (28.1)	96.8 (11.3)	0.05
Tryptophan					55.5 (12.6)	60.7 (9.6)	52.7 (9.2)	0.20

P-values are from linear regression models, comparing group means after adjustment for gender and age.

### Gastric and adipose hormones

We investigated fasting and postprandial serum levels of leptin and ghrelin. Fasting (06:00) ghrelin levels were higher in premanifest HD patients, but not in stage II/III HD patients, a borderline significant result (p = 0.051 for comparison between 3 groups, adjusted for age and gender). As with the amino acid data, this finding is unlikely have a biological underpinning and is consistent with chance. There was no significant difference in fasting (06:00) or postprandial (13:00) leptin or in postprandial (13:00) ghrelin levels between controls, premanifest and stage II/III HD subjects (Table 2).

## Discussion

Peripheral manifestations of Huntington’s disease such as weight loss and muscle wasting are thought to be linked to altered metabolism [2]. In this study we investigated metabolic alterations through evaluating circulating metabolic factors and functional tests. In contrast to many previous studies, we found little evidence to support alterations in metabolic markers with respect to disease progression. The differences in the findings between our study and previously published reports could be due to several reasons including cohort differences, sample handling and storage and differences in methodology. For instance, in our cohort we excluded patients on medication that could affect the hypothalamic-pituitary axis, and those who had experienced recent weight changes, which may have excluded some of the most severely affected HD patients. We also studied patients in a standardised way to control for exercise, diet and sleep.

### Glucose metabolism

Several studies suggest a high prevalence of glucose intolerance and diabetes mellitus in patients with HD [7, 8, 16, 17]. In addition, HD mouse models display pancreatic islet cell atrophy and intranuclear inclusions [57, 58]. Insulin sensitivity studies in HD patients show both a decrease in insulin sensitivity and impaired insulin secretion capacity in normoglycemic subjects [9]. Conversely, other studies in HD patients reported normal glucose and insulin levels following a glucose tolerance test, as well as normal fasting glucose and insulin [10–15]. Failure of GH suppression after glucose load [59], as well as an early GH and cortisol rise after an insulin tolerance test have been reported in HD [12, 60]. Adrenal cortical hypertrophy and increased cortisol levels were found in R6/2 mice and it was suggested that this resulted in reduced bone mineral density, skeletal muscle atrophy and insulin resistance [47].

In our study there was no significant difference in the insulin, growth hormone curve or cortisol secretion throughout the oral glucose tolerance test. There was no increased incidence of altered glucose homeostasis in patients with moderate and premanifest HD. The fact that patients with diagnosed diabetes were excluded in our study would not explain this lack of difference in glucose tolerance in patients without overt diabetes.

### Peripheral hormones: Leptin and Ghrelin

White adipose tissue, a peripheral endocrine organ, releases adipokines such as leptin and has a key role in energy metabolism and weight regulation. Leptin controls satiety, energy expenditure and neuroendocrine function through hypothalamic pathways [61, 62]. Leptin affects lipid metabolism, stimulates fatty acid oxidation and inhibits hepatic triglyceride accumulation [36].

Ghrelin is a gut peptide and is a natural ligand to growth hormone secretagogue receptor [34]. Produced by cells in the oxyntic glands of the stomach, it exerts a counter regulatory effect on leptin and it increases adiposity as it normally stimulates food intake and inhibits energy expenditure [36].

It has been suggested that HD patients are in a hypercatabolic state with negative energy balance [5, 63]. Endocrine links between the stomach, adipocytes and the brain that regulate energy intake and growth hormone release have been implicated in these processes. Popovic and colleagues (2004) found increased levels of ghrelin and decreased leptin levels in plasma from HD patients in non-fasting state, suggestive of negative energy balance [40]. Both ghrelin and leptin target hypothalamic neurons in the arcuate nucleus, ventromedial and lateral hypothalamus that express high levels of leptin and ghrelin receptors. The selective neuronal loss in lateral tuberal nucleus of hypothalamus may play a role in weight loss in early HD [4]. This rise in ghrelin levels and compensatory decrease in leptin levels may help to preserve body weight and maintain energy homeostasis. Our study is the first to look at fasting and postprandial leptin and ghrelin levels. Contrary to findings in other studies [50, 51, 64], our results did not show altered leptin or ghrelin levels, except for a borderline significant different in fasting ghrelin levels, with higher levels in premanifest (but not stage II/III) HD patients, which we believe is consistent with chance in view of the large number of analyses performed. It is possible that more convincing results would be obtained with a higher number of subjects. In keeping with these negative results, MRI of abdominal fat tissue did not show significantly altered adipose tissue depots in human subjects, in contrast to data from HD mouse studies [57]. Again, it might be that a larger subject cohort is needed, as the MRI data suggest that there might be a tendency towards altered proportion of subcutaneous versus in visceral fat in premanifest HD.

### Lipids

Lipids are vital to the health of the central nervous system, and research has revealed lipid dysregulation in HD [65]. This dysregulation has been linked to specific actions of mutant huntingtin on sterol regulatory element binding proteins, resulting in lower cholesterol levels in affected areas of the brain with evidence that this depletion is pathologic [66]. Studies on lipid metabolism have reported altered cholesterol and fatty acid metabolism in HD [66]. A metabolic study has demonstrated increases in various markers of fatty acid breakdown, including glycerol and malonate in HD gene carriers compared to controls [5]. High fasting concentrations of non-esterified fatty acids in HD patients have also been reported [18]. Dysfunction of the cholesterol biosynthetic pathway has been shown in peripheral fibroblasts and neurons of manifest HD patients [19, 20] and cholesterol precursors lanosterol and lathosterol, brain-derived cholesterol metabolite 24S-hydroxycholesterol, and bile acid precursor 27-hydroxycholesterol, were found to be significantly reduced in manifest HD patients [21, 22]. In our study, we did not observe difference in fasting serum cholesterol levels, however, we did not quantify levels of cholesterol metabolites.

### Proteins

Muscle wasting is a feature of manifest and pre-symptomatic HD. It is thought that the muscle wasting may be partly a result of impaired mitochondrial function and a local biochemical defect in HD muscles [67]. Amino acids are the building block of muscle and alterations in neutral amino acid metabolism in HD patients have been implicated for many years [18, 31]. The most consistent finding appears to be correlation between branched chain amino acid levels (particularly alanine, valine, leucine, isoleucine), weight loss, disease progression and abnormal triplet repeat expansion [5, 14, 18, 29–34]. In addition, considerable systemic alterations in the kinetics of the kynurenine pathway (a major route accounting for the metabolism of over 90% of the non-protein tryptophan in most tissues) have been reported in patients with HD [68]. Free plasma tryptophan levels were markedly reduced in HD subjects [18]. It was suggested that this represents dysregulation of energy expenditure and altered mitochondrial electron transport activity [5]. However, in our study there was no support for amino acid alteration in HD. Significant findings were found by HPLC for phenylalanine, lysine and arginine, but these would be consistent which chance based on the number of analyses performed in the study. The same is true for the tyrosine result using ion exchange chromatography and is supported by the fact that this result is different from the HPLC data. One difference between our study population and the population previously analysed [14] is that neither our premanifest individuals nor our HD patients had a lower BMI compared to controls. Instead, the BMI of HD gene carriers were higher, particularly in premanifest individuals.

### Bone turnover markers

The morphogenesis and remodelling of bone requires the synthesis of bone matrix by osteoblasts and its coordinated resorption by osteoclasts. Osteoprotegerin, is a key factor inhibiting the differentiation and activation of osteoclasts, and is, therefore, essential for bone resorption [69], whereas osteopontin is an important factor in bone remodelling [70]. Bone cells produce endocrine hormones that regulate glucose homeostasis [71] and osteocalcin, a bone-specific protein, was shown to regulate glucose metabolism in mice [72]. Reduced bone mineral density has been suggested to be an early feature of HD [73], suggesting that HD patients exhibit osteoporosis, probably as a direct effect of illness, due to the effect of mutant huntingtin on osteoclasts or osteoblasts in bone tissue or from immobility due to the disease. Bone turnover markers like osteocalcin, osteopontin and osteoprotegerin were measured in fasted state and compared to controls, pre-manifest and manifest HD patients. We did not show convincing evidence of abnormalities in the bone turnover markers measured, which is in keeping with findings we reported in a different cohort [74]. These data suggest that bone turnover markers are not useful as markers of bone turnover disturbances in HD.

### Limitations of this study

This study compared controls with premanifest and moderate HD patients in a standardised sampling period of 24 hours. Patients on neuroleptic and other medications, which are known to affect metabolic pathways, were excluded from this study, a design factor not included in many previous cohort studies. However, this does mean that patients who may have been more severely affected were excluded. Whilst we were unable to closely match premanifest and control group gender and age, we believe this is unlikely to alter our findings. Also, the premanifest subjects included in the study had a higher BMI compared to controls. It is possible that, because of high BMI, some of the metabolic dysregulation or compensatory mechanisms that take place due to the huntingtin mutation may be hidden. However, this would not explain lack of confirmation of metabolic changes in our stage II/III HD cohort. Another limitation of the study was the relatively small size of cohorts (13–15 subjects in each). This restricted the power to identify small to moderate effect sizes in variables. However, the careful controlling of medication, feeding and timing of sampling in this study meant that the groups were more easily comparable.

## Conclusion

We performed an extensive study focusing on metabolic factors including lipid and bone markers, glucose homeostasis, peripheral hormones affecting energy homeostasis and amino acids and their association with Huntington’s disease. In contrast to many previous studies, our results are consistent with no material differences between HD subjects and control, with just five statistically significant results (osteoprotegerin, tyrosine, phenylalanine, lysine and arginine levels), which are most likely to represent chance findings. The predominantly negative results suggest that the majority of these markers probably do not differ markedly by HD disease status, however a larger sample size is needed for more definitive evidence of smaller effects.

## Supporting Information

S1 Table Data set for demographic data in Table 1. (XLS)

S2 Table Data set for metabolic data in Table 2. (XLS)

S3 Table Data set for amino acids by ion exchange chromatography. (XLS)

S4 Table Data set for amino acids by HPLC. (XLS)
